# Breed-specific innate immune responses to *Brucella abortus* strain RB51 and Bacillus Calmette-Guérin in cattle

**DOI:** 10.1038/s41598-026-51050-8

**Published:** 2026-05-01

**Authors:** Haley M. Sterle, Bruna Petry, Mitchell V. Palmer, Steven C. Olsen, Paola M. Boggiatto, Ellie J. Putz

**Affiliations:** 1 National Animal Disease Center, Agricultural Research Service (USDA), Ames, IA USA; 2 Immunobiology Graduate Program, Iowa State University, Ames, IA USA; 3 Oak Ridge Institute for Science and Education, Oak Ridge, TN USA

**Keywords:** Diseases, Immunology, Microbiology

## Abstract

Bovine brucellosis and bovine tuberculosis are chronic diseases that cause economic and animal health concerns in the global beef and dairy industries due to production losses. The etiologic agents of these diseases, *Brucella abortus* and *Mycobacterium bovis*, are intracellular, zoonotic bacterial pathogens that pose a risk to human health. *Brucella abortus* strain RB51 (RB51) and Bacillus Calmette-Guérin (BCG) are live attenuated vaccines for bovine brucellosis and tuberculosis, respectively, and are known to reduce incidence of these diseases in cattle. However, recent work has suggested that the genetic background of cattle may influence immune responses to these two vaccines. Control of brucellosis and tuberculosis depends on T helper 1 (Th1) mediated immune responses; however, both RB51 and BCG are intracellular bacteria that predominantly localize in monocytes and macrophages which are responsible for initiating immune responses to disease. This study investigates the difference in responses of Hereford and Holstein monocyte-enriched peripheral blood mononuclear cells infected in vitro with RB51, BCG, and combined RB51 + BCG. Transcriptomic data was collected to conduct a targeted evaluation of differentially expressed genes between cell conditions from Holsteins and Herefords related to innate immune responses against intracellular bacteria and the downstream generation of adaptive immune responses. Additionally, breed-specific differences in the production of pro-inflammatory, anti-inflammatory, and Th1-related cytokines by infected monocyte-enriched cells were evaluated. After 16 h of infection with RB51, BCG, or RB51 + BCG, monocyte-enriched cells from Holsteins displayed a transcriptomic profile consistent with enhanced pro-inflammatory and Th1-polarizing responses compared to infected cells from Herefords. Further, Holstein cells also produced significantly higher amounts of Th1-polarizing cytokine than Hereford cells after 72 h of intracellular infection. The breed-specific differences in cellular responses presented here following in vitro infection with RB51, BCG, and RB51 + BCG provide evidence that Hereford and Holstein cattle may develop variable immune responses in vivo to these two vaccines. Results of this study suggest that vaccine efficacy may be influenced by cattle host genetic background within the same species and thus warrants consideration when optimizing vaccination strategies.

## Introduction

*Brucella abortus* and *Mycobacterium bovis* are the causative agents of bovine brucellosis and bovine tuberculosis (bTB), respectively. The World Organization for Animal Health (WOAH) classifies bovine brucellosis and bTB as diseases of importance due to their major economic impact on the global cattle industry and risk to public health as zoonotic diseases^1^. Bovine brucellosis causes reproductive failure and late-term abortions in cattle and other reservoir species. Transmission mainly occurs via ingestion or inhalation of bacteria from infected secretions and aborted tissues^2^. Animals with bTB infrequently present visible signs of infection^3^, however, the hallmark lesion of tuberculosis, the granuloma, can be found in the lungs and pulmonary lymph nodes of infected cattle^4^. Spread of bTB occurs when a secondary host ingests aerosolized bacteria expelled by an infected individual^5^.

The incidence of bovine brucellosis and tuberculosis is concentrated globally in developing countries. Though uncommon in the United States, co-infections with *B. abortus* and *M. bovis* in domestic cattle herds and wildlife have been reported in other countries including Canada, South Korea, Burkina Faso, and Nigeria^6–10^. The prevalence of co-infections has not been widely evaluated, but there is significant overlap in the geographic regions at risk for brucellosis and bTB^11–15^. Vaccination and test-and-slaughter programs have proven to be effective methods to reduce incidence of both diseases. However, implementation of these programs has varied worldwide due to their high cost and extensive labor requirements, as well as reintroduction of disease from wildlife reservoirs^16,17^. Reducing cases of brucellosis and bTB in livestock is important to minimize further economic losses in the cattle industry and prevent zoonoses.

*Brucella abortus* strain RB51 (RB51) is a live attenuated, commercially available bovine brucellosis vaccine that is highly effective at preventing abortions in cattle when administered prior to pregnancy between 4 and 12 months of age^2,18–20^. Because of its lack of an O antigen, RB51 does not interfere with serological diagnostic assays for brucellosis infection and thus is classified as a DIVA (Differentiation of Infected from Vaccinated Animals) vaccine^21^. Bacillus Calmette-Guerin (BCG) is a live attenuated strain of *M. bovis* that is known to be protective against bTB infections in cattle, reducing lesion severity in experimentally challenged animals^22–25^. However, unlike RB51, BCG vaccination can interfere with standard diagnostic testing^26^ and is not approved for commercial use in the United States. Despite efforts to develop other efficacious DIVA vaccines against *M. bovis*, BCG remains a primary option for a vaccine to control bTB^27^.

Though vaccination of cattle with RB51 and BCG ultimately generates adaptive T helper 1 (Th1) immune responses that are protective against virulent *B. abortus* and *M. bovis*, respectively^28,29^, macrophages also play a major role in the immune response to these intracellular bacteria. *Brucella* and *Mycobacteria* are recognized by pattern recognition receptors (PRR) on macrophages, triggering production of pro-inflammatory cytokines including tumor necrosis factor (TNF)-α, interleukin (IL)-1β, IL-6, and IL-12^30,31^. In the pro-inflammatory environment, macrophages polarize to an M1 phenotype^32,33^ and increase surface expression of co-stimulatory molecules involved in the process of antigen presentation to T cells^34^. Macrophages phagocytose and degrade bacteria, then present bacterial peptides on major histocompatibility class-II (MHC-II) to be recognized by CD4^+^ T cells^35,36^. Co-stimulation of T cells by CD80/86 on M1 macrophages provides a secondary activation signal^37^, and the presence of IL-12 in the proinflammatory environment polarizes antigen-specific CD4^+^ T cells towards a Th1 phenotype^38^. As virulent *Brucella* and *Mycobacteria* primarily infect host macrophages, Th1 cells capable of producing interferon (IFN)-γ are highly important for their ability to increase microbicidal activity of macrophages and enhance clearance of the intracellular bacteria^39,40^.

Development of a safe, efficacious, combined vaccine against *M. bovis* and *B. abortus* could help reduce cost and labor intensity of control efforts for both bovine brucellosis and bTB globally. Previous work by our group evaluating the feasibility of a combined vaccine yielded conflicting results. Initially, co-administration of RB51 and BCG to dairy-beef crossbred heifers increased the number of BCG-specific IFN-γ^+^ CD4^+^ T cells compared to vaccination with BCG alone, suggesting co-administration could potentially improve vaccine efficacy. Upon repetition of the experiment in Holstein steers, no difference in the number or functional capacity of BCG-specific CD4^+^ T cells was observed between cattle vaccinated with RB51 + BCG and BCG alone^41^. While numerous variables could contribute to differences between these studies, the difference in bovine host genetic background is a major candidate for consideration as a source of variation. Recent genomic evaluations of dairy cattle have shown that artificial selection pressure for milk production traits in the Holstein breed over approximately 60 years has inadvertently negatively affected unrelated genes, many related to immunity^42^. Importantly, in vitro RB51-induced transcriptomic responses were altered in contemporary Holstein cattle compared to animals with a genetic background representative of the breed prior to heavy artificial selection (i.e., unselected Holsteins)^43^. In contrast, beef producers select for a wide range of traits based on the unique goals of individual operations; therefore, beef cattle have not been subjected to the same directional artificial selection pressure as dairy cattle. Knowing that the genetic background of cattle may affect immune responses to live attenuated bacterial vaccines, we chose to compare in vitro responses to RB51 and BCG from two different breeds of cattle that represent beef and dairy production, Herefords and contemporary Holsteins.

In this study, we sought to investigate a range of innate immune parameters that may influence downstream adaptive immune responses to the RB51 and BCG vaccines in a representative beef and dairy breed. A combination of transmission electron microscopy, RNA sequencing, and cytokine production were used to thoroughly assess the innate immune profile of RB51, BCG, and RB51 + BCG infected monocyte-enriched PBMCs from Herefords and Holsteins. Our data provide novel information on the variability in immune responses to RB51 and BCG across the cattle industry. This study complements previous work to thoroughly describe immune responses to RB51 and BCG vaccination and contributes to our understanding of the variability in bovine innate immunity.

## Results

### Infection of monocytes with the intracellular bacteria RB51 and BCG

**Fig. 1:**
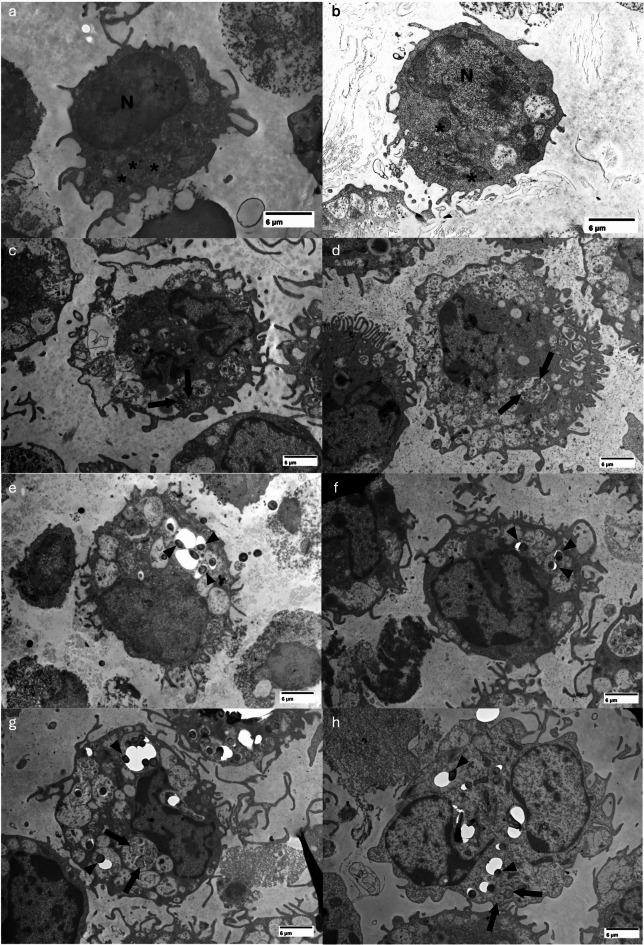
Representative transmission electron microscopy of monocytes infected with RB51 and/or BCG. (**a**, **b**) Uninfected monocytes from (**a**) Herefords and (**b**) Holsteins. Nuclei (N) and mitochondria (*) are of normal appearance. (**c**, **d**) Monocytes from (**c**) Herefords and (**d**) Holsteins infected with RB51 (black arrows), with RB51 bacteria in various stages of deterioration. (**e**, **f**) Monocytes from (**e**) Herefords and (**f**) Holsteins infected with BCG (black arrowheads). Empty space surrounding BCG bacteria is characteristic of imaged mycobacteria^44,45^. (**g**, **h**) Monocytes from (**g**) Herefords and (**h**) Holsteins co-infected with RB51 (black arrows) and BCG (black arrowheads). Magnification at 6800x.

Evaluation of monocyte-enriched samples by TEM confirmed intracellular infection of monocytes from Herefords and Holsteins with RB51 and BCG. Cell nucleus and mitochondria were identified in healthy, uninfected control monocytes from each breed (Fig. 1a,b). Monocytes from Herefords and Holsteins were readily infected by RB51 (Fig. 1c,d) and BCG (Fig. 1e,f). Representative images from each breed support the occurrence of co-infection of individual monocytes with both RB51 and BCG (Fig. 1g,h). Here, RB51 and BCG were observed in separate intracellular membrane-bound vacuoles in all samples from Herefords and Holsteins. BCG was distinguished from RB51 in co-infected cells by the distinct vacuole structure associated with mycobacteria^44,45^.

### Quantification of differential gene expression in monocyte-enriched cells

To gain insight on the effect of RB51 and BCG infection on monocyte gene regulation, we evaluated differential gene expression between infected and uninfected monocyte-enriched cells from Herefords and Holsteins. Initial analysis showed significant transcriptional changes due to bacterial infection with RB51, BCG, or RB51 + BCG in both breeds (Table 1). Out of the 37,063 annotated genes in the applied bovine genome, RB51, BCG, and RB51 + BCG infection in monocyte-enriched Hereford cells resulted in 6,435, 8,298, and 7,688 differentially expressed genes (DEG), respectively (p-adj < 0.05). The same infection conditions in monocyte-enriched cells from Holsteins induced differential expression of 5,202, 6,319, and 5,830 genes, respectively. All comparisons within the Hereford breed had more DEG than the corresponding comparisons in Holsteins.

**Table 1:** Total number of differentially expressed genes (DEG) and corresponding number of upregulated and downregulated genes between contrasts of interest. Upregulated genes in within-breed comparisons (Hereford x Hereford and Holstein x Holstein) represent higher expression under infection Condition B, while downregulated genes represent higher expression under infection Condition A. Upregulated genes in between-breed comparisons (Hereford x Holstein) represent higher expression in Holstein monocytes, while downregulated genes represent higher expression in Hereford monocytes under the same infection condition.

Condition A	Condition B	Total DEG	# Upregulated Genes	# Downregulated Genes
*Hereford x Hereford*				
Uninfected	RB51	6435	3280	3155
Uninfected	BCG	8298	4157	4141
Uninfected	RB51 + BCG	7688	3925	3763
RB51	BCG	1237	309	928
RB51	RB51 + BCG	388	72	316
BCG	RB51 + BCG	2	2	0

Condition A	Condition B	Total DEG	# Upregulated Genes	# Downregulated Genes
*Holstein x Holstein*				
Uninfected	RB51	5202	2617	2585
Uninfected	BCG	6319	3051	3268
Uninfected	RB51 + BCG	5830	2905	2925
RB51	BCG	90	27	63
RB51	RB51 + BCG	16	7	9
BCG	RB51 + BCG	0	0	0

Condition	Total DEG	# Upregulated Genes	# Downregulated Genes
*Hereford x Holstein*			
Uninfected	1797	849	948
RB51	1160	704	456
BCG	2591	1651	940
RB51 + BCG	1981	1233	748

Additionally, the effect of breed on gene regulation was evaluated by comparing monocyte-enriched cells from Herefords and Holsteins that were subjected to the same infection condition. Even when comparing uninfected cells, the two breeds exhibited different transcriptional signatures (Table 1). A total of 1,797 significant DEG were observed, of which 849 were upregulated in monocyte-enriched Holstein cells and 948 were upregulated in monocyte-enriched Hereford cells (i.e., downregulated in monocyte-enriched cells from Holsteins). RB51 infection saw a reduced number of DEG between breeds to 1,160, while infection with BCG and RB51 + BCG showed 2,591 and 1,981 significant DEG, respectively. In all three infected cell comparisons, more genes were upregulated in monocyte-enriched cells from Holsteins than from Herefords.

### Differential expression of selected monocyte-associated genes

To further explore the effects of RB51, BCG, and RB51 + BCG on the innate immune profile of monocyte-enriched cells from Herefords and Holsteins, we specifically investigated 25 genes of interest based on their relevance to intracellular bacterial infection, monocyte/macrophage immune response, and polarization of Th1 vs Th2 responses. Monocyte-enriched cells from both breeds infected with RB51, BCG, and RB51 + BCG significantly increased transcription of proinflammatory cytokine genes *IL1B*, *IL6*, and *TNF* compared to uninfected cells (Table 2). When evaluating proinflammatory gene expression between breeds, higher proinflammatory transcriptional signatures were observed in monocyte-enriched cells from Holsteins than from Herefords (Table 2). Transcripts for subunits p35 and p40 of the Th1 polarizing cytokine IL-12^46^, encoded by *IL12A* and *IL12B*, respectively, were notably unchanged between uninfected and infected monocyte-enriched cells from Herefords. In contrast, *IL12A* and *IL12B* were both upregulated during infection of Holstein cells. Further, *IL12B* was upregulated in monocyte-enriched Holstein cells compared to Hereford cells in response to RB51, BCG, and RB51 + BCG infection (Table 2). We assessed *IL18* due to its ability to enhance Th1 responses by promoting IFN-γ production^47^, and found that *IL18* was downregulated in response to RB51, BCG, and RB51 + BCG in both breeds (Table 2). Transcriptional expression of a Th2-polarizing cytokine gene, *IL4*^48^, was unchanged following infection of monocytes-enriched cells from both breeds (Table 2). Expression of chemokine genes *CCL3*, *CCL4*, *CCL5*, and *CXCL10* increased broadly in response to RB51, BCG, and RB51 + BCG in both breeds, but significantly more so in monocyte-enriched cells from Holsteins than from Herefords (Table 2).

**Table 2:** Differential expression of selected cytokine and chemokine genes between uninfected and infected monocyte-enriched cells from Herefords (Hereford x Hereford) and Holsteins (Holstein x Holstein), and between Hereford and Holstein cells under the same infection condition (Hereford x Holstein).

Gene	*Uninfected x RB51*		*Uninfected x BCG*		*Uninfected x RB51* + *BCG*	
	Log2FC	adj p-value	Log2FC	adj p-value	Log2FC	adj p-value
*Hereford x Hereford*						
*IL1B*	7.2522	1.12E-18	7.3617	1.88E-20	7.4832	2.95E-21
*IL4*	*NS*	*NS*	*NS*	*NS*	*NS*	*NS*
*IL6*	8.2107	4.03E-93	7.0455	7.18E-59	7.4744	1.73E-65
*IL10*	4.0199	1.71E-144	3.9723	6.07E-200	4.2375	2.06E-225
*IL12A*	*NS*	*NS*	*NS*	*NS*	*NS*	*NS*
*IL12B*	*NS*	*NS*	*NS*	*NS*	*NS*	*NS*
*IL13*	5.2132	1.12E-09	5.3806	1.27E-10	5.3739	1.77E-10
*IL18*	− 0.9073	7.65E-04	− 1.3312	9.56E-08	− 1.3499	1.21E-08
*IL23A*	1.6186	1.01E-06	2.4629	1.81E-23	2.3934	1.72E-18
*TNF*	4.0730	1.27E-45	4.3580	3.06E-116	4.4120	2.32E-101
*CCL3*	6.0127	3.43E-31	6.0271	2.01E-42	6.1871	7.37E-41
*CCL4*	7.9198	1.03E-17	7.7293	1.83E-18	7.9596	4.14E-19
*CCL5*	3.8643	5.42E-34	4.2931	1.95E-81	4.4633	7.58E-71
*CXCL10*	*NS*	*NS*	5.9228	1.08E-32	6.0868	2.78E-33

Gene	*Uninfected x RB51*		*Uninfected x BCG*		*Uninfected x RB51* + *BCG*	
	Log2FC	adj p-value	Log2FC	adj p-value	Log2FC	adj p-value
*Holstein x Holstein*						
*IL1B*	7.6431	1.15E-39	7.7860	4.61E-42	7.8946	1.88E-40
*IL4*	*NS*	*NS*	*NS*	*NS*	*NS*	*NS*
*IL6*	8.1801	1.23E-61	6.8760	8.77E-46	7.2460	1.12E-45
*IL10*	4.0528	2.05E-52	3.9296	1.32E-35	4.2279	1.00E-31
*IL12A*	2.6800	3.21E-03	*NS*	*NS*	2.0978	3.32E-02
*IL12B*	4.6211	1.28E-09	4.8059	7.91E-15	5.0902	4.39E-14
*IL13*	5.3930	2.48E-08	5.5689	6.68E-08	5.4695	2.55E-10
*IL18*	− 1.4337	3.01E-04	− 1.6869	8.77E-07	− 1.7157	3.17E-06
*IL23A*	3.2212	5.52E-11	3.8687	1.13E-18	3.9122	5.64E-17
*TNF*	5.2982	1.90E-105	5.6096	3.02E-92	5.7093	1.22E-90
*CCL3*	5.8825	1.00E-67	6.0551	4.36E-61	6.2472	4.39E-46
*CCL4*	8.1247	3.46E-78	8.1295	8.04E-73	8.3666	1.80E-63
*CCL5*	4.6258	1.91E-34	5.2416	1.41E-39	5.5488	6.88E-28
*CXCL10*	6.6374	9.64E-09	6.8110	1.36E-11	6.6765	1.47E-11

Gene	*Uninfected x Uninfected*		*RB51 x RB51*		*BCG x BCG*		*RB51* + *BCG x RB51* + *BCG*	
	Log2FC	adj p-value	Log2FC	adj p-value	Log2FC	adj p-value	Log2FC	adj p-value
*Hereford x Holstein*								
*IL1B*	*NS*	*NS*	0.8463	1.56E-02	0.8668	6.84E-04	0.8638	1.75E-03
*IL4*	*NS*	*NS*	*NS*	*NS*	*NS*	*NS*	*NS*	*NS*
*IL6*	*NS*	*NS*	0.9274	1.91E-02	0.7778	1.21E-02	*NS*	*NS*
*IL10*	*NS*	*NS*	*NS*	*NS*	*NS*	*NS*	*NS*	*NS*
*IL12A*	*NS*	*NS*	*NS*	*NS*	*NS*	*NS*	*NS*	*NS*
*IL12B*	*NS*	*NS*	2.1895	1.24E-03	1.6129	1.08E-04	1.7342	1.41E-04
*IL13*	*NS*	*NS*	*NS*	*NS*	*NS*	*NS*	*NS*	*NS*
*IL18*	*NS*	*NS*	*NS*	*NS*	*NS*	*NS*	*NS*	*NS*
*IL23A*	*NS*	*NS*	1.0706	2.91E-02	1.2896	1.23E-02	1.1884	1.69E-02
*TNF*	*NS*	*NS*	0.8780	2.94E-02	0.8833	2.08E-03	0.9419	2.75E-03
*CCL3*	*NS*	*NS*	*NS*	*NS*	1.1881	2.43E-04	1.2266	1.11E-02
*CCL4*	*NS*	*NS*	1.0516	1.40E-02	1.2357	2.23E-04	1.2504	6.05E-03
*CCL5*	*NS*	*NS*	*NS*	*NS*	1.0989	4.94E-03	1.2420	4.15E-02
*CXCL10*	*NS*	*NS*	*NS*	*NS*	*NS*	*NS*	*NS*	*NS*

In single-breed comparisons, positive Log2 fold change represents increased gene expression in infected cells, while negative Log2 fold change represents decreased gene expression in infected cells. In Hereford x Holstein comparisons, positive Log2 fold change represents increased gene expression in Holstein cells, while negative Log2 fold change represents increased gene expression in Hereford cells. NS indicates differential gene expression was not significant (adj p-value > 0.05).

An enzymatic gene marker of the M1 phenotype and the respiratory burst reaction, inducible nitric oxide synthase (*NOS2*)^49^, was significantly upregulated in monocyte-enriched cells from both Herefords and Holsteins in response to RB51, BCG, and RB51 + BCG (Table 3). When comparing uninfected cells from each breed, we found that *NOS2* was upregulated in monocyte-enriched cells from Holsteins (p = 0.0102, Log2FC = 1.5777) (Table 3). M1-associated surface marker transcripts of *CD40*, *CD80*, and *TNFSF9*^34,50,51^ were increased upon infection with RB51, BCG, and RB51 + BCG in monocyte-enriched Hereford and Holstein cells. In contrast, gene expression of another M1-associated surface marker, CD86^34^, was unchanged or downregulated in response to RB51, BCG, and RB51 + BCG in monocyte-enriched cells from both breeds (Table 3). Differential expression of the M2-associated enzyme gene *ARG1*^52^ was absent regardless of breed or infection condition (Table 3). Expression of the gene for CD163, a scavenger receptor closely associated with an M2 phenotype^52^, was downregulated significantly following all infection conditions in both breeds (Table 3). *BOLA-DRB2*, a bovine MHC-II associated gene^53^, was the only targeted gene significantly upregulated in monocyte-enriched cells from Herefords (Table 3).

**Table 3:** Differential expression of selected M1 and M2 associated genes between uninfected and infected monocyte-enriched cells from Herefords (Hereford x Hereford) and Holsteins (Holstein x Holstein), and between Hereford and Holstein cells under the same infection condition (Hereford x Holstein).

Gene	*Uninfected x RB51*		*Uninfected x BCG*		*Uninfected x RB51* + *BCG*	
	Log2FC	adj p-value	Log2FC	adj p-value	Log2FC	adj p-value
*Hereford x Hereford*						
*NOS2*	6.5051	1.41E-86	8.5781	1.06E-163	8.4315	1.71E-168
*ARG1*	*NS*	*NS*	*NS*	*NS*	*NS*	*NS*
*SOD2*	4.4139	5.04E-36	3.9673	5.93E-18	4.1413	1.64E-19
*NOX5*	*NS*	*NS*	*NS*	*NS*	*NS*	*NS*
*CD14*	− 0.6139	9.89E-04	− 1.5653	1.46E-20	− 1.3158	7.96E-20
*CD40*	3.1479	2.53E-32	2.3897	1.49E-26	2.7222	4.25E-32
*CD80*	0.7844	3.37E-05	1.0143	6.02E-10	0.8927	9.91E-08
*CD86*	− 1.1780	2.31E-10	− 2.2137	1.82E-52	− 2.0377	3.37E-40
*CD163*	− 2.7727	3.59E-17	− 5.6268	1.27E-163	− 5.0296	4.32E-110
*TNFSF9*	3.8474	9.86E-39	3.9710	1.28E-40	3.9692	1.19E-39
*BOLA-DRB2*	0.6623	1.92E-02	0.8600	6.41E-03	1.0911	3.73E-04

Gene	*Uninfected x RB51*		*Uninfected x BCG*		*Uninfected x RB51* + *BCG*	
	Log2FC	adj p-value	Log2FC	adj p-value	Log2FC	adj p-value
*Holstein x Holstein*						
*NOS2*	7.2324	6.01E-56	8.0528	1.24E-71	8.0652	2.76E-67
*ARG1*	*NS*	*NS*	*NS*	*NS*	*NS*	*NS*
*SOD2*	4.8626	4.49E-27	4.5066	4.98E-23	4.6642	2.08E-22
*NOX5*	*NS*	*NS*	*NS*	*NS*	*NS*	*NS*
*CD14*	− 0.7084	1.69E-02	− 1.3515	1.26E-06	− 1.2044	3.33E-05
*CD40*	3.3688	1.36E-22	2.9106	2.10E-18	3.1319	6.24E-16
*CD80*	0.6915	2.31E-02	0.8542	1.88E-03	0.8232	1.20E-02
*CD86*	*NS*	*NS*	− 1.3169	1.66E-07	− 1.0567	1.54E-03
*CD163*	− 2.5574	6.00E-08	− 5.0106	3.21E-33	− 4.5594	1.50E-27
*TNFSF9*	3.9001	5.58E-21	4.2904	7.64E-25	4.2819	1.04E-22
*BOLA-DRB2*	*NS*	*NS*	*NS*	*NS*	1.0401	3.34E-02

Gene	*Uninfected x Uninfected*		*RB51 x RB51*		*BCG x BCG*		*RB51* + *BCG x RB51* + *BCG*	
	Log2FC	adj p-value	Log2FC	adj p-value	Log2FC	adj p-value	Log2FC	adj p-value
*Hereford x Holstein*								
*NOS2*	1.5777	1.02E-02	2.3252	1.18E-09	1.0636	1.01E-02	1.2306	3.00E-03
*ARG1*	*NS*	*NS*	*NS*	*NS*	*NS*	*NS*	*NS*	*NS*
*SOD2*	*NS*	*NS*	0.7356	1.72E-02	0.8141	7.88E-03	0.8080	2.30E-02
*NOX5*	*NS*	*NS*	3.5648	1.51E-03	2.3404	3.06E-02	2.5887	8.72E-04
*CD14*	*NS*	*NS*	*NS*	*NS*	0.7021	1.96E-02	0.6093	4.80E-02
*CD40*	*NS*	*NS*	*NS*	*NS*	0.7843	2.02E-03	*NS*	*NS*
*CD80*	*NS*	*NS*	*NS*	*NS*	*NS*	*NS*	*NS*	*NS*
*CD86*	*NS*	*NS*	0.9243	1.88E-02	0.8009	1.51E-03	0.9006	1.42E-02
*CD163*	*NS*	*NS*	*NS*	*NS*	*NS*	*NS*	*NS*	*NS*
*TNFSF9*	*NS*	*NS*	1.2786	3.91E-06	1.5350	6.70E-07	1.5381	7.20E-06
*BOLA-DRB2*	*NS*	*NS*	*NS*	*NS*	-0.9735	1.96E-02	-0.9875	3.30E-02

In single-breed comparisons, positive Log2 fold change represents increased gene expression in infected cells, while negative Log2 fold change represents decreased gene expression in infected cells. In Hereford x Holstein comparisons, positive Log2 fold change represents increased gene expression in Holstein cells, while negative Log2 fold change represents increased gene expression in Hereford cells. NS indicates differential gene expression was not significant (adj p-value > 0.05).

Collectively, differential expression of the 25 genes of interest in uninfected monocyte-enriched cells from Herefords and Holsteins indicated similar resting transcriptional profiles between cells derived from the two breeds (Tables 2, 3). However, we observed a broad upregulation of innate immune genes in monocyte-enriched cells from Holsteins compared to Herefords in response to infection with RB51, BCG, and RB51 + BCG (Tables 2, 3). This suggests that cells from Holsteins display an enhanced activation state for genes related to immune responses to intracellular bacterial infection as compared to cells from Herefords.

### Ingenuity pathway analysis of differentially expressed genes between monocyte-enriched cells from Herefords and Holsteins

**Fig. 2:**
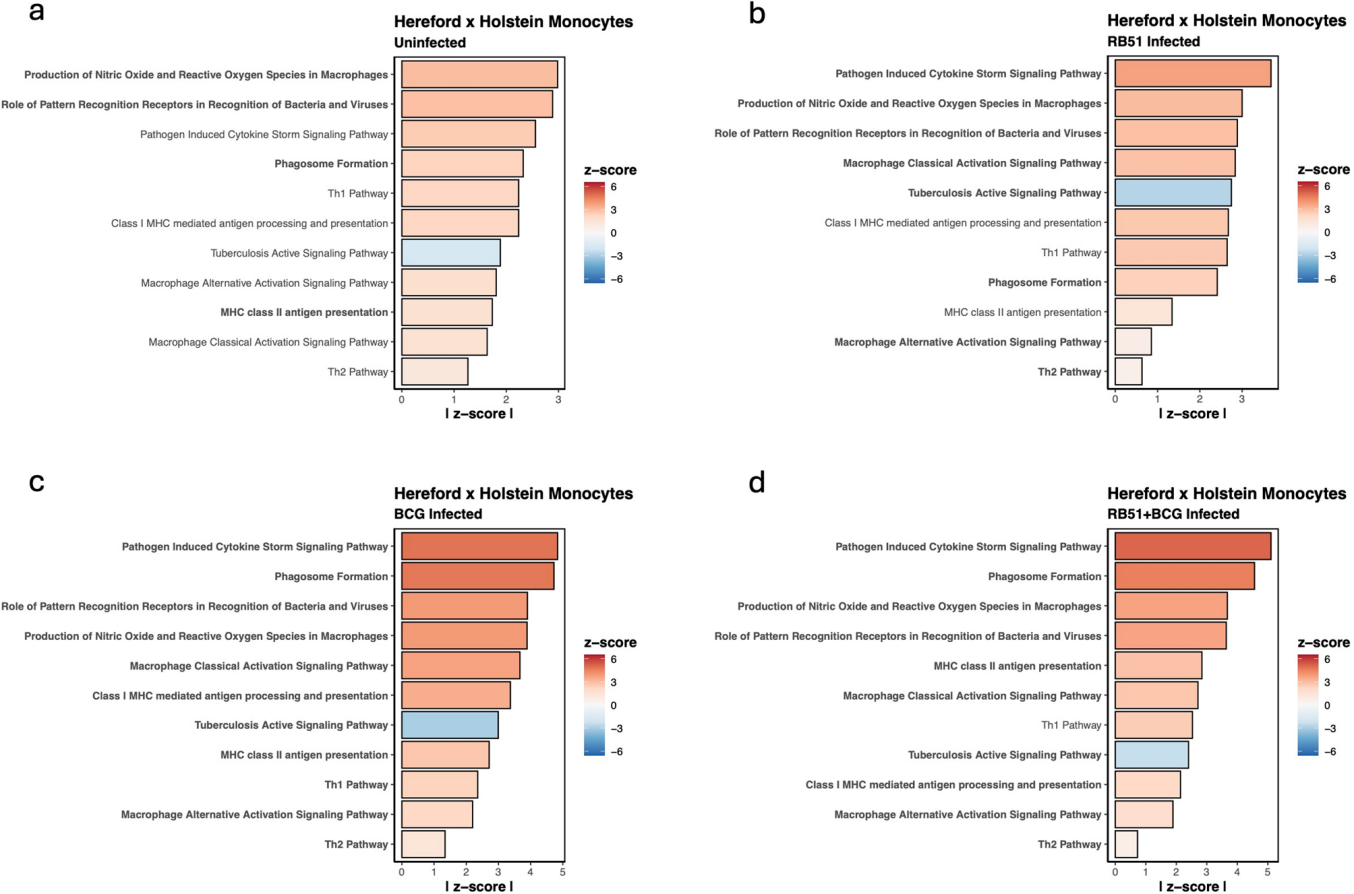
Ingenuity Pathway Analysis evaluation of all DEG between Hereford and Holstein monocyte-enriched cells. Comparisons are between (**a**) uninfected cells, (**b**) RB51 infected cells, (**c**) BCG infected cells, and (**d**) RB51 + BCG infected cells. Positive z-scores indicates a positive association with pathway term in Holstein monocytes, whereas a negative z-score indicates positive association with pathway term in Hereford monocytes. Pathway terms in bold text are significant (p ≤ 0.05).

To further analyze the differences in the transcriptional profiles between Herefords and Holsteins, we used the Ingenuity Pathway Analysis (IPA) software to evaluate 11 canonical pathways related to innate immunity against intracellular pathogens and the subsequent induction of an adaptive immune response. We compared pathways of uninfected, RB51, BCG, and RB51 + BCG infected monocyte-enriched cells from Herefords and Holsteins. When comparing uninfected cells from both breeds, we found a significant increase in terms related to the production of nitric oxide (NO) and reactive oxygen species (ROS) in macrophages, recognition of bacteria and viruses by pattern recognition receptors (PRR), phagosome formation, and antigen presentation by MHC-II in monocyte-enriched cells from Holsteins compared to Herefords (Fig. 2a, bold pathways). This is consistent with the upregulation of *NOS2* in uninfected monocyte-enriched Holstein cells reported above.

RB51 infection promoted a transcriptomic profile associated with cytokine storm signaling, macrophage-specific responses to pathogens, and the Th2 pathway in monocyte-enriched Holstein cells (Fig. 2b). Infection with BCG and RB51 + BCG significantly increased signatures associated with 10/11 and 9/11 pathways, respectively, in monocyte-enriched Holstein cells as compared to Hereford cells (Fig. 2c,d). Interestingly, the tuberculosis active signaling pathway was the lone term significantly increased in monocyte-enriched cells from Herefords in response to RB51, BCG, and RB51 + BCG infection (Fig. 2b-d). Collectively, compared to Herefords, the Holstein transcriptomic profiles of RB51, BCG, and RB51 + BCG conditions were highly associated with innate immune-related canonical pathways, suggesting that monocyte-enriched cells from Holsteins broadly exhibit enhanced immune activity following intracellular bacterial infection when compared to Hereford cells.

### Expression of inflammatory and Th1-associated cytokines by infected monocyte-enriched cells

**Fig. 3:**
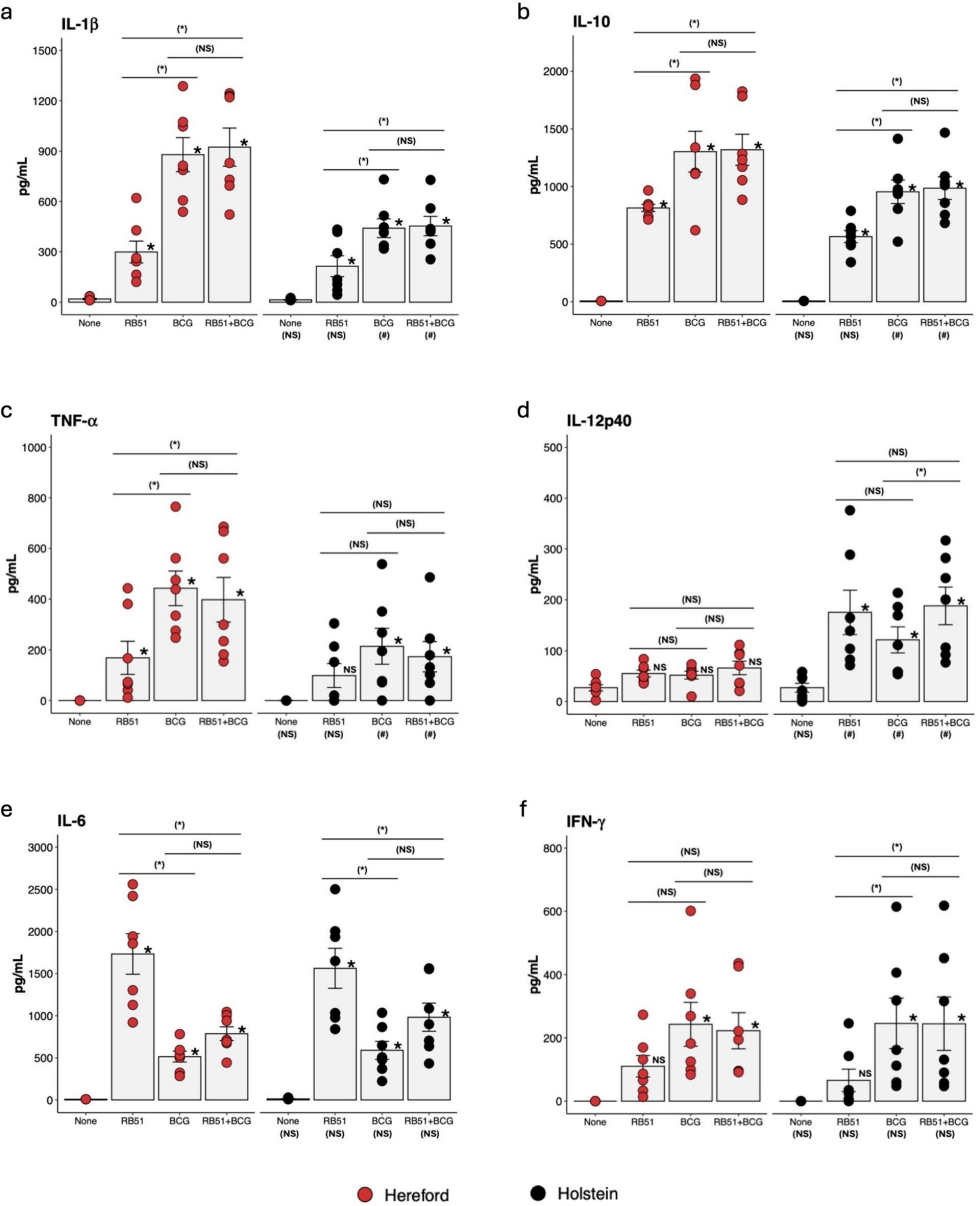
Concentrations of proinflammatory, anti-inflammatory, and Th1-associated cytokines after 72 h of monocyte-enriched cell infection. Mean concentration (gray bars) of (**a**) IL-1β, (**b**) IL-10, (**c**) TNF-α, (**d**) IL-12p40, (**e**) IL-6, and (**f**) IFN-γ by uninfected (None), RB51 infected, BCG infected, or RB51 + BCG infected monocyte-enriched cells from Herefords (red circles) and Holsteins (black circles). NS or * on upper corner of mean bar indicates significance between unstimulated cells from the same breed. (NS) or (*) indicates significance between RB51 and BCG, BCG and RB51 + BCG, or RB51 and RB51 + BCG infected cells. (NS) or (#) below x axis indicates significance in cytokine production between Hereford and Holstein cells of the same infection group. Circles represent individual animal cytokine concentrations and gray bars represent group means ± SEM. *NS*, no significance; * or #, *p* ≤ 0.05.

Differences in the Hereford and Holstein monocyte-enriched cellular responses to RB51, BCG, and RB51 + BCG infection were also investigated by evaluating the production of cytokines IL-1β, IL-6, IL-10, IL-12p40, TNF-α, and IFN-γ after in vitro infection. Monocyte-enriched cells from Herefords produced higher quantities of IL-1β, IL-10, and TNF-α in response to BCG and RB51 + BCG infection compared to monocyte-enriched cells from Holsteins (p < 0.02), though no differences between breeds were observed with RB51 infection alone (Fig. 3a-c). In response to all three infection conditions, Holstein monocyte-enriched cells produced more IL-12p40 than Hereford monocyte-enriched cells (p < 0.04) (Fig. 3d). In fact, infection of Hereford monocyte-enriched cells did not induce production of IL-12p40 under any infection condition as compared to uninfected cells (p > 0.2). Cattle breed had no effect on the production of IL-6 or IFN-γ, as infection with RB51, BCG, and RB51 + BCG resulted in production of similar levels of these cytokines by monocyte-enriched cells from Holsteins and Herefords (Fig. 3e-f). Taken together, the pattern of cytokine production from monocyte-enriched Hereford cells was representative of an increased inflammatory response to intracellular bacteria when compared to cytokines produced by Holstein cells. In contrast, the higher production of IL-12p40 by monocyte-enriched Holstein cells suggests generation of an enhanced Th1-polarizing environment in response to intracellular bacteria compared to Hereford cells.

## Discussion

Previous work evaluating the effect of combining RB51 and BCG vaccines on the bovine adaptive immune response suggests that cattle breed may play a role in variation of the immune response induced following vaccination and/or co-vaccination^41^. Here, we sought to further characterize the immune responses to RB51 and BCG in two breeds of cattle with distinct genetic backgrounds, representing the two main production systems, beef (Herefords) and dairy (Holsteins). We conducted a targeted evaluation of in vitro responses of blood-derived, monocyte-enriched cells from Hereford and Holstein cattle infected with RB51, BCG, and RB51 + BCG. The data presented here illustrate breed-specific variability in initial responses to live attenuated bacterial vaccines from two common cattle breeds in the US, highlighting an area of further research to better serve both dairy and beef producers.

In our in vitro system, transmission electron microscopy indicated that blood-derived monocytes from cattle have the capacity to internalize RB51 and BCG. When RB51 and BCG are co-administered, we demonstrate that both bacteria can be internalized into separate intracellular vacuoles within the same monocyte regardless of cattle breed. This implies that in vivo, monocyte activation could be influenced by internal signaling from numerous vaccine agents. Follow up work should investigate the capacity of differentiated and tissue resident macrophages to concurrently uptake RB51 and BCG in vivo when vaccines are combined and administered simultaneously and any subsequent effects on macrophage signaling.

Investigation into the transcriptomic signatures of monocyte-enriched cells first revealed that compared to uninfected cells, infection with RB51, BCG, or RB51 + BCG resulted in differential expression of upwards of 14% of the annotated genes within the bovine genome regardless of cattle breed. To better assess early responses to RB51 and BCG, we performed a focused, in-depth evaluation of differential expression of monocyte-related genes associated with recognition of and response to intracellular bacteria. Accordingly, we observed an upregulation of genes encoding proinflammatory cytokines and chemokines, enzymes involved in the respiratory burst, and M1-associated surface markers after RB51, BCG, and RB51 + BCG infection of monocyte-enriched cells from both Herefords and Holsteins. Further, and not surprisingly given the nature of the organisms used in the study, evidence of significant M2- and Th2-polarized responses to bacterial infection was lacking. Notably, no changes in *IL4* or *ARG1* expression were observed and *CD163* expression was downregulated in response to infection with RB51, BCG, and RB51 + BCG in monocyte-enriched cells from both Herefords and Holsteins. These data suggested that monocyte-enriched cells from both breeds are capable of recognizing and initiating transcriptional changes congruent with exposure to intracellular bacteria.

When comparing the transcriptional response between breeds, we were able to identify numerous breed-vs.-breed differences in gene expression related to immune function. Monocyte-enriched cells from Holsteins showed significant upregulation of proinflammatory cytokine genes *IL1B*, *IL6*, and *TNF*, and proinflammatory chemokine genes *CCL3*, *CCL4*, and *CCL5*^54^ compared to Hereford monocyte-enriched cells. Additionally, we observed increased expression of *NOS2* and *NOX5*, two genes associated with the respiratory burst^49,55^, in Holstein monocyte-enriched cells. Collectively, these observations indicate that while cells from both breeds demonstrate a proinflammatory and anti-microbial transcriptional profile in response to intracellular bacterial infection, these functions appear to be enhanced in monocyte-enriched Holstein cells when compared to monocyte-enriched Hereford cells.

As with expression of cytokine, chemokine, and respiratory burst-associated enzyme genes, we observed that monocyte-enriched Holstein cells exhibited increased expression of *CD40*, *CD86*, and *TNFSF9* as compared to monocyte-enriched Hereford cells. CD40, CD86, and 4-1BBL (encoded by *TNFSF9*) interact with CD40L, CD28, and 4-1BB on T cells, respectively, during antigen presentation^50,56,57^. Co-receptor/ligand interactions during antigen presentation activate both T cells and macrophages, stimulating proliferation and IL-2 production by T cells and inducing the pro-inflammatory and antimicrobial activity of macrophages^58–60^. Upregulation of co-receptors and ligands involved in antigen presentation in monocyte-enriched Holstein cells suggests that Holstein cells may have an enhanced co-stimulatory activity compared to Hereford cells when infected with intracellular bacteria, which may translate to a stronger interaction between innate and adaptive immunity in Holstein cattle.

Our analysis also included evaluation of monocyte-associated genes important for the initiation and polarization of Th1 responses to intracellular bacteria, which are critical to host control of disease in vivo. Interestingly, we found that compared to uninfected cells, expression of *IL12A* (IL-12p35) and *IL12B* (IL-12p40) did not change in monocyte-enriched Hereford cells infected with RB51, BCG, or RB51 + BCG. In contrast, evaluation of infected monocyte-enriched cells from Holsteins did show upregulation of *IL12A* and *IL12B* compared to uninfected cells, but only in conditions that included RB51, as infection with BCG alone only increased expression of *IL12B*. Furthermore, the *IL12B* transcriptomic data was corroborated by cytokine quantification showing that significant increases in IL-12p40 protein were only observed in supernatants from monocyte-enriched Holstein cells following RB51, BCG, and RB51 + BCG infection. As the classical Th1-polarizing cytokine, IL-12 plays a critical role in stimulating the production of IFN-γ by CD4^+^ T cells, which can then increase microbicidal activity of macrophages to combat intracellular bacteria^38,61^. Murine studies have suggested that secretion of IL-12 in response to *B. abortus* infection is directly responsible for inducing IFN-γ production^62^, and that the protective effect of RB51 vaccination against virulent challenge is augmented by IL-12^63^. Similar observations regarding the importance of IL-12 have been made for BCG. Treatment of murine splenocyte cultures with anti-IL-12 significantly reduces IFN-γ production after BCG exposure^64^, and conversely, co-vaccination of mice with BCG and a plasmid encoding IL-12 enhances both IFN-γ production and protection against virulent challenge compared to BCG alone^65^. We could hypothesize that in cattle, increased amounts of bioactive IL-12 generated by RB51 and BCG vaccination may provide enhanced protection against virulent infection. However, in our in vitro system, production of IL-12p40 by only infected monocyte-enriched Holstein cells did not result in differential IFN-γ cytokine production between cells from Holsteins and Herefords. Altogether, these data suggest that monocyte-enriched cells from Holsteins may potentially have an enhanced ability to generate a Th1-polarizing environment in response to intracellular bacteria, though the biological significance and effect of variable Th1-polarizing cytokine production on breed-specific adaptive immune responses have yet to be determined.

Due to the importance of adaptive immune responses in the control of intracellular bacteria, we evaluated additional parameters associated with generation of cell-mediated immunity. Expression of the *IL18* gene along with two downstream indicators of a Th1 response, IFN- γ production and gene expression of the chemokine gene *CXCL10*^66^, show no differences between monocyte-enriched cells from Herefords and Holsteins. Likewise, there was no difference in expression of the Th2-associated cytokine gene *IL13*^67^ or anti-inflammatory cytokine gene *IL10*^68^ between infected cells derived from the two different breeds. Finally, transcriptomic profiles associated with Th1 and Th2 pathways were only moderately different between infected cells from Holsteins and Herefords. In our study, the 16- and 72-h infection time points were geared toward early evaluation of innate immune parameters. Further investigation into the Th1- and Th2-polarizing environment at later time points would better capture breed-specific contrasts in the generation of an adaptive immune response to RB51 and BCG beyond the observed differences in IL-12 at the time points analyzed here.

Collectively, the transcriptomic data presented in this study suggests that innate immune cells from Holstein cattle broadly exhibit enhanced immune activation in response to intracellular bacteria as compared to innate immune cells from Hereford cattle. However, this may not translate to increased protection against bacterial infections in Holsteins compared to Herefords. Evaluating differences in immune function between contemporary Holsteins and Holsteins unselected for milk production traits since the 1960’s has shown that in the context of an experimental intramammary *Escherichia coli* challenge model, contemporary animals exhibit a stronger inflammatory and more rapid immune response than unselected cows, both systemically and in the mammary tissue^69^. However, contemporary cattle displayed greater indicators of disease severity in response to the *E. coli* infection than did unselected cattle. Unselected Holsteins also controlled bacterial infection more effectively than contemporary Holsteins, which suggests that the enhanced immune response in unselected cattle was unproductive for controlling infection^69^. Thus, the broadly increased innate immune activation in Holstein cells compared to Hereford cells observed at the time points analyzed here, as measured by differential gene expression and IPA terms, may not correspond to a beneficial in vivo host response to intracellular bacteria.

Interestingly, in monocyte-enriched cells from both breeds, the in vitro responses to infection with RB51 + BCG and BCG alone were much more alike than were the responses to infection with RB51 + BCG and RB51 alone. The similarity in the transcriptomic profile of cells infected with BCG alone and RB51 + BCG was highlighted in the canonical pathway analysis between monocyte-enriched cells from Herefords and Holsteins. Notably, the association of infected monocyte-enriched Hereford cells and the IPA term “tuberculosis active signaling pathway” suggests that Hereford innate immune cells may exhibit a transcriptomic response consistent with control of intracellular *Mycobacterium*. In cell supernatants, we saw that infection with BCG alone and RB51 + BCG induced different concentrations of IL-12p40 in monocyte-enriched cells from Holsteins but not Herefords. Production of all other cytokines was the same between intracellular infection with BCG alone and RB51 + BCG. Collectively, this indicates that the response to infection of monocyte-enriched cells with RB51 + BCG may be driven predominantly by BCG and suggests that co-vaccination of cattle with RB51 + BCG may result in a BCG-dominated immune response.

Collectively, this preliminary study describes novel differences in the innate immune response between breeds of a beef and dairy background and highlights avenues for future work. We acknowledge that monocyte-enriched cultures contained PBMCs other than pure monocytes, and that investigating the responses of pure and differentiated macrophage populations would be beneficial. We also recognize that using PBMCs from Holstein steers and Hereford heifers may have introduced a sex effect into the results described herein. Further exploration into the kinetics of transcriptomics and protein expression could benefit from direct time point comparisons and additional infection periods. Lastly, the in vitro nature of this study, coupled with the limited pre-existing comparisons between the host genetic background of the Hereford and Holstein breeds, prevents us from making definite conclusions on the biological significance of the observed breed-specific differences.

The data presented here provide evidence of breed-specific variation in the bovine innate immune response to intracellular bacteria both by gene regulation and cytokine production. Broadly, we observed the upregulation of genes associated with innate immunity and monocyte activation in monocyte-enriched Holstein cells infected with RB51, BCG, and RB51 + BCG compared to Hereford cells. Transcriptomic profiles further suggest that even in the absence of stimuli, innate immune function in Holsteins may be inherently enhanced compared to Herefords. Further, the differences in Th1-polarizing cytokine production between breeds indicates potential variability in the development of adaptive immune responses. The full biological significance of the breed-specific differences observed in our in vitro study, and how these differences apply directly to beef and dairy production systems, has yet to be determined. Additional beneficial work would include comparisons between individual sorted cell types and total PBMC preparations, and ideally, in vivo characterization of responses to intracellular bacteria in a wide range of cattle breeds. However, the preliminary work presented here suggests that there may be a need to optimize live attenuated bacterial vaccination strategies to provide optimal efficacy in both beef and dairy cattle. We are invested in continuing this work to expand the current limited understanding of how divergent genetic backgrounds affect immune responses in cattle and to inform best practices in animal health.

## Materials and methods

### Animal care

All cattle used in this study were obtained from a bovine brucellosis and bovine tuberculosis free herd (Superior Livestock, Inc., Joice, IA). The two groups of cattle in this study were two-year-old Hereford heifers and two-year-old Holstein steers (n = 7 each). Breeds were housed outdoors in separate field barns on the National Animal Disease Center (NADC) campus in Ames, Iowa. All housing and blood collection procedures were designed in accordance with the Guide for the Care and Use of Agricultural Animals in Research and Teaching and were approved prior to the study by the NADC Animal Care and Use Committee.

### Peripheral blood mononuclear cell (PBMC) isolation

Whole blood was collected from Hereford heifers and Holstein steers via jugular venipuncture into acid citrate dextrose (ACD) anticoagulant and PBMCs were isolated as previously described^28^. Live cell counts were determined for each sample using Trypan blue exclusion and counting on a hemocytometer. Cell suspensions were adjusted to a final concentration of 1 × 10^7^ cells per mL in complete 1640 RPMI (cRPMI) medium (Cat. No. 11875119, Thermo Fisher Scientific, Waltham, MA) containing 20% heat-inactivated FBS (Cat. No. SH30070.03, Cytiva, Wilmington, DE), 1% HEPES (Cat. No. 15630080, Thermo Fisher Scientific), 1% non-essential amino acids (Cat. No. M7145, Sigma-Aldrich, Burlington, MA), 1% essential amino acids (Cat. No. M5550, Sigma-Aldrich), 1% sodium pyruvate (Cat. No. S8636, Sigma-Aldrich), 100 U/ml penicillin (Cat. No. 15140122, Thermo Fisher Scientific), 100 μg/ml streptomycin (Cat. No. 15140122, Thermo Fisher Scientific), 2 mM L-glutamine (Cat. No. G7513, Sigma-Aldrich), and 50 μM 2-beta mercaptoethanol (Cat. No. M6250, Sigma-Aldrich).

### Blood-derived monocyte enrichment and in vitro infection

To enrich for blood-derived monocytes, PBMCs were plated onto either 6- or 24-well plates at 8 × 10^6^ or 4 × 10^6^ per well, respectively, in cRPMI medium. Plates were incubated overnight at 37 °C with 5% CO_2_ to allow adherent cells to attach. Nonadherent cells were removed the next morning by gently dumping off medium. Wells used for supernatant collection were washed gently with warm cRPMI. Prior to the study, average monocyte yield for Herefords and Holsteins were determined after adherent cell enrichment using Trypan blue staining and counting on a hemocytometer. Plating 8 × 10^6^ PBMC in a 6-well plate yielded approximately 1–1.5 × 10^6^ monocytes per plate from both breeds, or 2.0–3.1% of total plated PBMCs. For study calculations, it was assumed that monocyte yield after adherent cell enrichment was 2% of total plated PBMCs.

Adherent monocyte-enriched cells were left uninfected (provided warm cRPMI only) or were infected with RB51 (Colorado Serum Company, Denver, CO), BCG Danish Strain 1331 (NADC, Ames, IA), or RB51 + BCG at a target multiplicity of infection (MOI) of 5:1. RB51 + BCG cells were infected with equal numbers of each bacteria (i.e., MOI of 2.5:1 for each). Plates were then incubated at 37 °C with 5% CO_2_ for various assays as detailed below.

### Transmission electron microscopy (TEM)

After 16 h of infection, medium was removed from monocyte-enriched cultures and adherent cells were washed gently with room temperature (RT) Dulbecco’s phosphate buffered saline (DPBS) (Cat. No. 14190136, Thermo Fisher Scientific). Plates were placed on ice for 10 min (min) and wells were scraped and pipetted gently for cell removal. Cells were collected and pelleted by centrifugation at 300×*g* for 10 min at RT. Cells were washed once more in DPBS, centrifuged as above, and fixed in 1 mL of 5% glutaraldehyde in 0.1 M Cacodylate buffer, pH 7.4. Samples were submitted to the NADC Microscopy Services lab where fixed samples were washed in the same buffer three times, embedded in 2% agar, and processed through graded alcohols prior to embedding in LR White resin (Electron Microscopy, Hatfield, PA)^70^. Ultrathin sections for electron microscopy were obtained and stained with 5% uranyl acetate and Reynold’s lead stain. Sections were examined on a ThermoFisher FEI Tecnai G^2^ BioTWIN electron microscopy (Hillsboro, OR). Images were taken with a side mount ORCA-HR digital camera (Advanced Microscopy Techniques, Woburn, MA).

### RNA isolation and sequencing

For RNA isolation, cells were collected as above after 16 h of infection. Cells were pelleted by centrifugation at 300×*g* for 10 min at RT. Pellets were resuspended in 1 mL TRIzol (Cat. No. 15596026, Thermo Fisher Scientific) and RNA isolation was conducted as previously described^71^. Briefly, the TRIzol suspension was incubated at RT for 10 min. Following, 260 μL chloroform was added, and samples were shaken vigorously at RT for 10 min. Phase separation was achieved by centrifuging tubes at 12,000×*g* for 15 min at 4 °C, and the aqueous phase was collected. Next, 660 μL isopropanol was added to the aqueous phase, mixed gently, and incubated at RT for 10 min. RNA was pelleted by centrifugation at 12,000×*g* for 10 min at 4 °C and the supernatant was removed. Pellet was washed with 1 mL 75% ethanol and recovered via centrifugation at 7,500×*g* for 5 min at 4 °C. Supernatant was decanted and pellet was air dried for approximately 30 min at RT to remove remaining ethanol. The RNA pellet was resuspended in 50 μL RNase-free water and incubated at 55 °C for 10 min to solubilize the RNA. Cleanup to remove residual DNA was conducted using the TURBO DNA-*free*™ Kit (Cat. No. AM1907, Thermo Fisher Scientific) according to manufacturer’s DNase treatment protocol. Final RNA concentration was quantified using a Qubit™ 4 Fluorometer (Thermo Fisher Scientific) and the Qubit RNA High Sensitivity Assay Kit (Cat. No. Q32855, Thermo Fisher Scientific) according to manufacturer’s recommendations.

RNA was submitted to the Roy J. Carver Biotechnology Genomics Center, University of Illinois (Urbana-Champaign, IL). Sample quality was confirmed by bioanalyzer, and library processing was performed using the Illumina mRNA kit. Samples were sequenced on the Nova Seq X Plus, generating 150 base pair paired-end reads.

### RNA-seq data processing and differential expression analysis

The RNA-seq data was processed using Nextflow^72^ (v24.04.2) with the nf-core/rna-seq pipeline^73^ (v3.14.0). Read quality was assessed using FastQC^74^ (v0.12.1), and adapter trimming was performed with TRIMgalore^75^ (v0.6.7), excluding reads with a Phred-score below 20. Post-filtering, an average of 70 million reads per sample were successfully mapped (mapping quality > 0), with summary metrics stats reported by samtools^76^ (v1.17) via MultiQC^77^ (v1.19). Sequence alignment was conducted against the *Bos taurus* reference genome (ARS-UCD2.0, NCBI) using STAR software^78^ (v2.7.10a) and gene-level counts were generated using featureCounts^79^ (v2.0.4).

Differential gene expression analysis was performed using DESeq2^80^ (v1.44.0) in R. Genes were filtered out if they had zero counts across all samples, lacked expression (i.e., zero reads) in more than three samples, or showed low overall abundance—defined as an average expression below three reads per sample across all samples. DE comparisons were conducted between Hereford and Holstein groups across infection groups (uninfected, BCG infected, RB51 infected and RB51 + BCG infected). Ingenuity Pathway Analysis^81^ (IPA; QIAGEN, Redwood City, CA, USA) was used to perform the core analysis and predict activation or inhibition of canonical metabolic pathways based on the resulting expression profiles.

### Quantification of cytokine production

Adherent monocyte-enriched cells were infected as detailed above. After 72 h of infection, 24-well plates were centrifuged at 300×*g* for 10 min at RT to clarify supernatants. Supernatants were then removed by pipette and stored at − 20 °C until analysis. AlphaLISA Immunoassay Kits (Revvity, Waltham, MA) were used to quantify concentrations of IL-1β, IL-6, IL-10, IL-12p40, TNF-α, and IFN-γ in supernatants from RB51, BCG, and RB51 + BCG infected and uninfected monocyte-enriched cultures (Cat. No. AL539HV, AL538HV, AL546HV, AL553HV, AL534HV, AL535HV, respectively). Individual kit protocols were followed, and Alpha signal was read using a Synergy Neo2 multi-mode microplate reader (Agilent BioTek, Santa Clara, CA) under standard Alpha detection settings. Standard curves were generated using GraphPad PRISM (version 10.5.0, Boston, MA) and data were analyzed using a 4-parameter logistic equation and 1/Y^2^ data weighting with values at maximal concentrations of analyte after the hook point removed when necessary. Concentrations of cytokines in supernatants were interpolated using the appropriate nonlinear regression equation.

Cytokine data were analyzed using a simple linear regression model in R (version 4.2.0). Cattle breed, infection condition, and the interaction between cattle breed and infection condition were set as fixed effects for all data. Pairwise comparisons of Least Squares Means were conducted to determine significance between specific contrasts of interest, with statistical differences identified when p-value ≤ 0.05. Data are presented as mean ± SEM.

## Data Availability

The RNA sequencing data generated and analyzed during this study are available at SRA-NCBI repository under accession number PRJNA1294790 at https://www.ncbi.nlm.nih.gov/bioproject/1294790.
